# De novo transcriptome assembly and comprehensive assessment provide insight into fruiting body formation of *Sparassis latifolia*

**DOI:** 10.1038/s41598-022-15382-5

**Published:** 2022-06-30

**Authors:** Lili Shu, Miaoyue Wang, Hui Xu, Zhiheng Qiu, Tianlai Li

**Affiliations:** School of Horticulture, Shenyang Agricultural University, Shenyang, 110866 China

**Keywords:** Computational biology and bioinformatics, Developmental biology, Microbiology, Environmental sciences, Molecular medicine, Astronomy and planetary science

## Abstract

The genes associated with fruiting body formation of *Sparasis latifolia* are valuable for improving mushroom breeding. To investigate this process, 4.8 × 10^8^ RNA-Seq reads were acquired from three stages: hyphal knot (SM), primordium (SP), and primordium differentiation (SPD). The de novo assembly generated a total of 48,549 unigenes, of which 71.53% (34,728) unigenes could be annotated by at least one of the KEGG (Kyoto Encyclopedia of Genes and Genomes), GO (Gene Ontology), and KOG (Eukaryotic Orthologous Group) databases. KEGG and KOG analyses respectively mapped 32,765 unigenes to 202 pathways and 19,408 unigenes to 25 categories. KEGG pathway enrichment analysis of DEGs (differentially expressed genes) indicated primordium initiation was significantly related to 66 pathways, such as “Ribosome”, “metabolism of xenobiotics by cytochrome P450”, and “glutathione metabolism” (among others). The MAPK and mTOR signal transduction pathways underwent significant adjustments during the SM to SP transition. Further, our research revealed the PI3K-Akt signaling pathway related to cell proliferation could play crucial functions during the development of SP and SPD. These findings provide crucial candidate genes and pathways related to primordium differentiation and development in *S. latifolia*, and advances our knowledge about mushroom morphogenesis.

## Introduction

Cauliflower mushroom (*Sparassis*) is widely known for its characteristic fruiting body that appears similar to cauliflower, and is recognized as an edible mushroom with numerous medicinal properties as well^1^. Among members of this genus, the only species that current can be artificially cultivated is *S. latifolia*. In the last several years, however, there has been successful progress on the artificial breeding of *Sparassis* in Japan, China, and Korea^2^. Prior to 2006, *S. latifolia* had been wrongly recognized as *S. crispa* until phylogenic evidence from three nuclear rDNA marker genes and mitochondrial gene confirmed *S. latifolia* as a new species^3^. Furthermore, the *S. crispa* of Asia present significant differences from conspecific strains originating from Europe and North America, so the Asian isolates were re-classified as *S. latifolia* based on a morphological comparison and molecular phylogeny analysis^4^.

Our current understanding of the process entailing fruiting body initiation and development is nascent, and how the mycelium forms the fruting body is a prominent subject in the field of fungi molecular biology, especially concerning *Coprinopsis cinerea*, *Schizophyllum commune*, and certain non-model species, such as *Agaricus bisporus*, *Flammulina velutipes*, and *Boletus edulis*^5,6^. The formation stage of fruiting body is the most complicated transformations in the cyclogeny of mushrooms, co-regulated by genetic, physiological, growth, as well as environmental factors^7^.

Due to the lack of knowledge about the key mechanism underpinning fruiting body formation, numerous edible mushrooms still cannot be readily cultivated via artificial techniques. For some valuable and rare mushroom species, particularly *S. latifolia*, little research had been conducted on how the developing fruiting body is controlled because suitable analytical tools and the prerequisite genetic information were unavailable^8^. Yang et al.found that vitamin B6 metabolism, glycine metabolism, and cystathionine lyase, which might play an important role during light-inducedprimordia formation^9^. Accordingly, a much better understanding of this process should enable researchers to realize the fruiting body formation of *S. latifolia*, potentially providing new opportunities to promote its industrial production.

Reverse and forward genetics tools such as RNAi and mutants analysis, have been widely applied to the research of gene function in many fungi^10–12^^.^ Among the four successive stages of mushroom formation, that is the hyphal knot, initial primordium, primordium, and fruiting body^13^, the formation of initial primordium is the most critical step. For different stages, some genes have been cloned whose functional analysis has been carried out. A recent report showed that *Nox* functioned as signal in crosstalk between reactive oxygen species (ROS) and the Ca_2_^+^ pathway, which regulated development of the mushroom fruiting body^14^. It has also been found that the *dst* gene is essential to transform primordia into mature fruiting bodies^15,16^. In *C. cinereus,* mutation of *Ubc2* could result in a fruiting defect by seriously affecting clamp cell morphogenesis and nuclear migration^17^, and *eln2* has been show to control the elongation of primordia^18,19^. Along with those genes, numerous transcription factors (TFs) might participate in the transition of asexual propagation to sexual propagation development, such as *WC-1*^20^, *FlbB*^21^, and *Pro1*^22^. Additionally, the MAPK and cAMP/PKA signaling pathways might be involved in regulating sexual development in fungi^23,24^.

Lately, rapid progress in sequencing technology, especially RNA sequencing, had undoubtedly advanced research in life sciences and substantially augmented our capacity for gene mining and functional analysis^25–28^. For example, the well-studied transcriptome of *C. cinerea* from its mycelium to primordium phase showed that transcriptomic changes lead the transformation from an undifferentiated structure to fruiting body structure^25,27^. Similar research was also performed in *A. bisporus*, *Agrocybe aegerita*, and *Hypsizygus marmoreus*, and much knowledge about mycelium-fruiting transition has been gained^28–31^. Yet, the mechanism responsible for the initiation of primordium and following developmental stages of *S. latifolia* remains understudied, hindering a comprehensive understanding of its fruiting development.

To fill this knowledge gap, here the transcriptome of three key developmental stages of *S. latifolia* was comparatively studied, to produce comprehensive sets of gene transcripts and glean critical information about transcriptome-level changes that occur as the fruiting body forms. This study can improve our understanding of dynamic changes characterizing the formation of the fruiting body, and point to some molecular mechanisms underlying *S. latifolia*’s development. Moreover, these first comprehensive transcripts sets of *S. latifolia* could serve as important resources for investigating other aspects of fruiting body formation more generally.

## Methods

### Sample preparation and RNA extraction

The *S. latifolia* strain (No. CCMJ 1100) was provided by the Culture Collection Center of Mycology of Jilin Agriculture University. Liquid spawn of the strain were inoculated into pine sawdust medium that had been sterilized at 121 °C for 2.5 h, and incubated in the dark at 24 °C. The pine sawdust medium contained 76% pine sawdust, 18% bran, 2% corn flour, 1.5% sucrose, 1.5% gypsum and 1% calcium superphosphate. The mycelia spread throughout the cultivation bags at 10 days post-inoculation (dpi) and began to form the hyphal knot, which could be visible at 70 dpi. The hyphal knots were collected and denoted as SM. The cultivation bags were then placed under controlled conditions (18–20 °C, 10 h/14 h light–dark cycle, light intensity maintained at 800–1000 Lux). After 100 dpi, a little primordium could be observed and this was sampled and denoted as SP. Later, at about the 115 dpi, the young fruiting body had formed and this was collected and denoted as SPD. For three distinct developmental stages (SM, SP, SPD), every treatment group consisted of three replicates. All samples were isolated carefully and promptly frozen in liquid nitrogen, then stored at − 80 °C.

Total RNA extraction of each sample was done using the TRIzol kit (Invitrogen, CA, USA), by following to its manual’s descriptions. Then, we checked the quality of RNA using agarose gel electrophoresis and a BioAnalyzer (Agilent, CA, USA), and measured the quantity of RNA using a NanoDrop spectrophotometer (Thermo Fisher Scientific, Waltham, Ma, USA). Eventually, a more than adequate amount of good quality RNA samples were obtained and these utilized for the RNA-Seq library construction and the quantitative real-time PCR analysis.

### Library construction and Illumina Sequencing

To construct the cDNA library and perform deep sequencing, the Illumina’s protocols were followed (Illumina Inc., San Diego, CA, USA). Briefly, DNA was degraded with DNase I, and the mRNA enriched with magnetic Oligo (dT). The mRNA was sheared into short fragments (280 bp) by a fragmentation buffer and these ensuing fragments served as templates to synthesize the first-strand cDNA using random hexamer primers synthesis; the second-strand was synthesized with DNA polymerase I and RNaseH (Thermo Fisher Scientific). Then deep sequencing was conducted on the Illumina Hi-Seq 2500 platform with the PE150 approach, by the Shanghai Realgene Biotechnology Corporation. Every sample was performed in three independent sequencing libraries. A total of 9 RNA-seq paired-end libraries were constructed from three samples (SM, SP, SPD).

### Transcriptome assembly of protein coding genes and their annotation

The raw paired-end reads were checked using the FastaQC Package (http://www.bioinformatics.babraham.ac.uk/projects/fastqc/). Clean reads were obtained after first removing their adapter, any low quality bases, and the short reads (< 20 bp), then we de novo assembled the transcriptome by using Trinity software (version Trinityrnaseq_r2014-04–13, http://trinityrnaseq.sourceforge.net/) under its default parameters^32^. A clustering analysis was implemented with CD-HIT software (version 4.8.1, https://github.com/weizhongli/cdhit)^33^, with a 95% identity threshold applied to minimize redundancy. The contigs were overlapped and aligned and these defined as the final sets of unigenes.

To predict their function, every unigene was assessed using Transdecoder software (version 5.5.0, http://transdecoder.github.io/), and then these were submitted as BLASTP queries, with an E-value < 1e^-5^, against four databases: GeneBank NR, Swiss-Prot, KOG, and KEGG^34^. GO annotation was done using Blast2GO^35^. Goatools (https://github.com/tanghaibao/Goatools) and KOBAS were utilized to perform the enrichment analysis for GO terms and KEGG pathways, respectively^36^.

### Identification and annotation of differentially expressed genes (DEGs)

The DEGs between two samples were isolated using the RSEM package^37^ and NOIseq (non-parametric approach for the differential expression analysis of RNA-Seq data in R) package (http://bioinfo.cipf.es/noiseq). RSEM was used to count the reads of every gene, whose level of gene expression quantified according to fragments per kilobase of exon per million mapped reads (FPKM)^38^. We used the |log_2_(fold change)|≥ 1 and probability ≥ 0.8 as the cut-off criteria to designate the DEGs. The GO and KEGG analyses of functional enrichment were performed against the whole-transcriptome background, with a Bonferroni corrected P-value set at 0.01.

### qRT-PCR analysis

The M-MLV reverse transcriptase (NEB, MA, USA), with oligo (dT), was used as the primer to reverse transcribe each sample of total RNA (1 μg). The genes of interest were respectively analyzed by qRT-PCR. Three biological replicates with three technical replicates were performed with 18S rRNA gene (*18sRNA*) as the internal control. The primers of selected genes (and *18sRNA*) are listed in Table S1. The SYBR Premix ExTaq kit (Takara, Dalian, China) was used, following the manufacturer’s recommendations, to perform the amplification. For this, the cycling parameters were 95 °C for 30 s, then 40 cycles of 95 °C for 5 s followed by 60 °C for 1 min. All samples were run in triplicate. The 2^-ΔΔct^ methodology, using its recommended parameters (FAST 7500, Agilent Technologies, CA, USA), was applied to quantify relative gene expression.

## Results

### Developmental stages of *S. latifolia*

**Figure 1:**
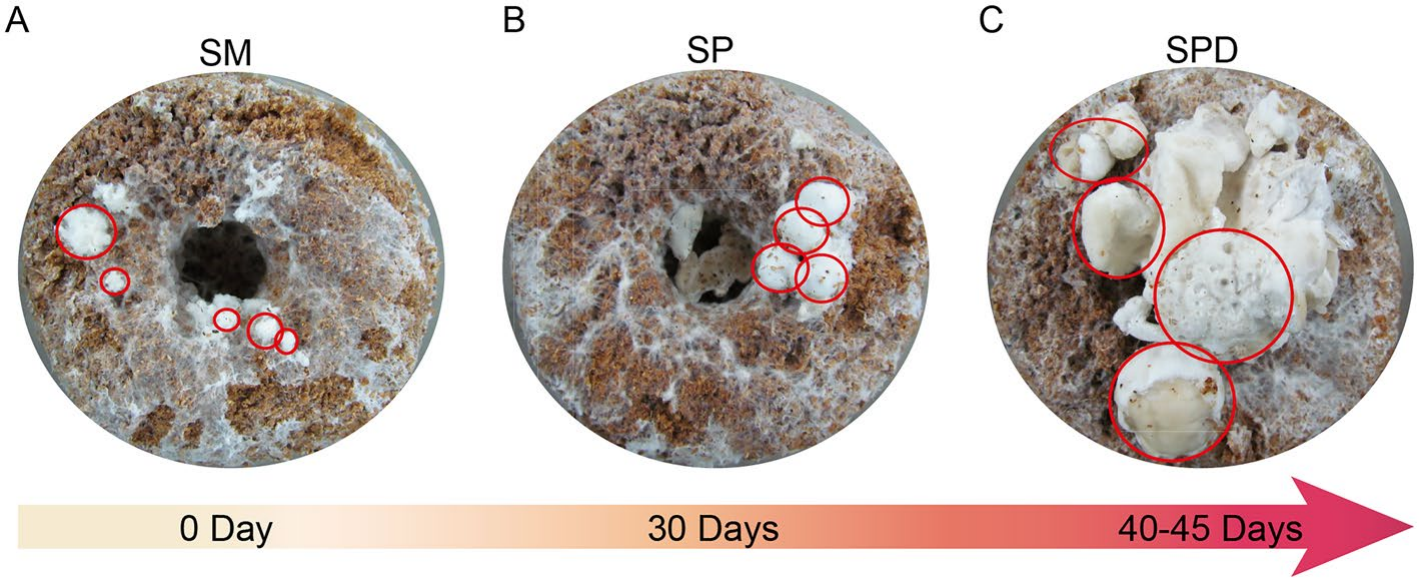
The developmental stages of *S. latifolia*. (**A**) the hyphal knot (SM); (**B**) primordium (SP); (**C**) primordium differentiation (SPD). The tissues of different stages are marked in red circles.

The development of *S. latifolia* could be divided into four successive and overlapping stages: the hyphal knot (SM), primordium (SP), primordium differentiation (SPD) (Fig. 1), and the final fruiting body stages. The transitional transformations from SM to SP, and from SP to SPD, are the most critical steps in cauliflower mushroom cultivation. Under the standard culture condition, the mature mycelium turned into hyphal knots (Fig. 1A), and the day of this event marked as 0 days after hyphal knots’ formation was clearly visible (DMKA); from this moment onward, the mycelium come into sexual reproduction. Each cultivation sample was then placed under a lit condition, and their hyphal knots gradually increased in both volume and density, accompanied by a burgeoning primordium after 30 DMKA (Fig. 1B). During primordium differentiation, many irregular acanthine bulges appear on the surface of each primordium during the 40–45 DMKA period (Fig. 1C).

### RNA sequencing and transcriptome assembly

Before the assembly, raw data was subjected to quality control to remove bad and low quality reads. The three samples (SM, SP and SPD) generated more than 60 Gb of clean data from paired-end (PE) reads, each sample comprised of three biological replicates (Table S2). Unigenes of different stages were assembled and integrated together by utilizing the software Trinity and CD-hit, which generated 48,549 unigenes (Table 1); their respective length mainly ranged from 300 to 4500 bp, and averaged 2488 bp (Fig. S1). When the clean reads were mapped back to the unigenes’ sets, most positions of the expressed genes were covered by more than 10^7^ reads (Fig. S2).

**Table 1:** The assembled results for *S. latifolia* transcriptome.

Unigenes		Number	Mean length (bp)	N50 (bp)	Reads mapped (%)
Stage	Replicate				
SM	1	23,415	1372	1904	99.4
	2	24,013	1415	1993	98.8
	3	23,921	1448	2,030	99.4
SP	1	25,101	2013	2930	99.2
	2	25,226	1936	2789	99.1
	3	24,807	1933	2755	98.9
SPD	1	24,456	2098	3035	99.2
	2	23,704	1883	2737	98.4
	3	24,460	2056	2978	99.2
Total	48,549	2488	3629	99.1	

### Functional annotation of the *S. latifolia* transcriptome

**Figure 2:**
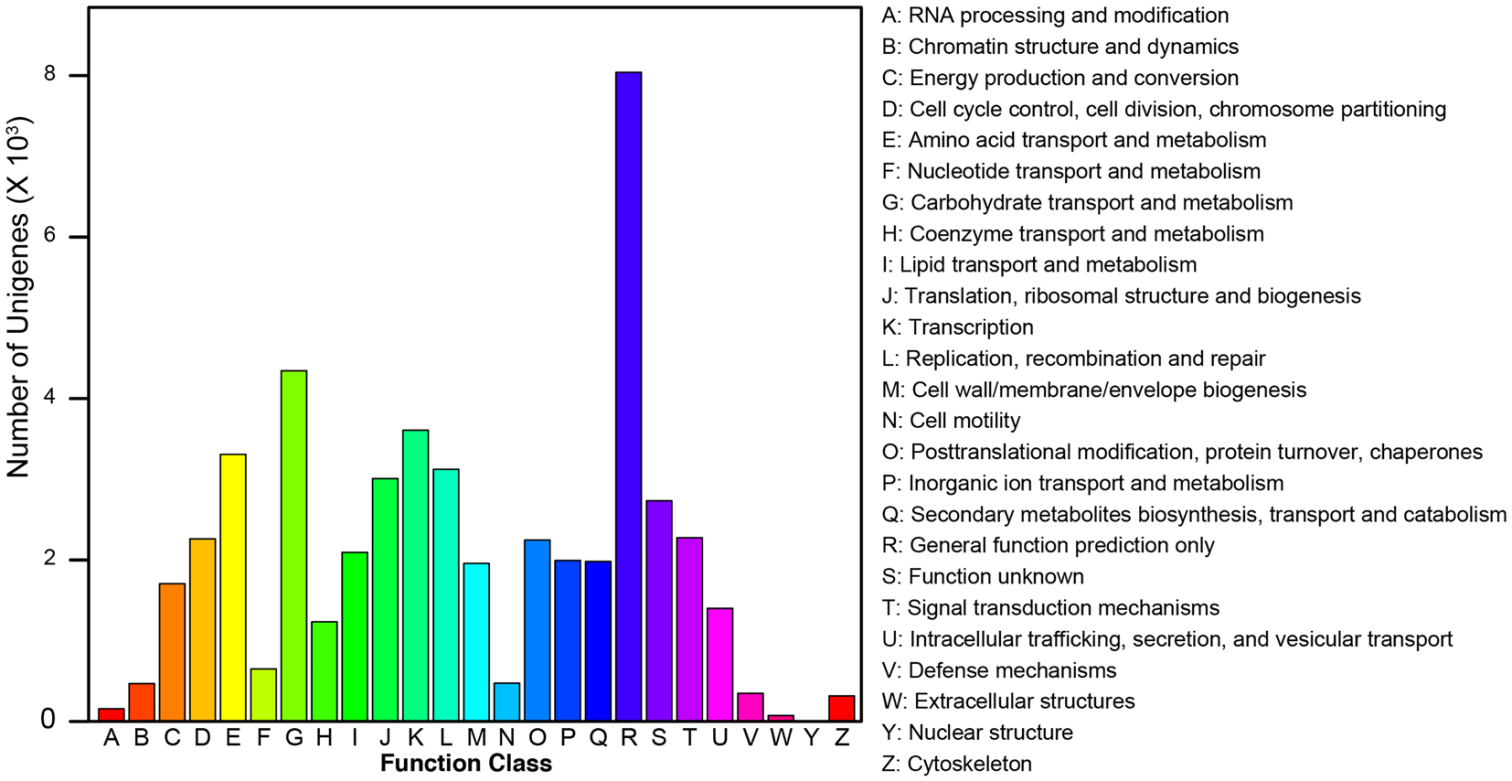
The KOG functional categories of *S. latifolia* unigenes.

By making use of TransDecoder, overall, 40,876 (84.19%) protein coding unigenes were obtained. These protein coding unigenes were annotated using BLASTP against the Nr protein database, of which 16.08% did not have any hits, and might instead be non-coding RNA transcripts. After counting the results for the Nr database search (Fig. S3, Dataset 1), we found that the top seven-hit fungi species were all basidiomycetes (Fig. S3), whose sets of unigenes matched well with the genes of *Trametes versicolor*, implying their close phylogenetic relationship. KOG classifications put 19,408 unigenes (46.6% of total protein coding unigenes) into 25 functional groups (Fig. 2 and Dataset 2). The largest cluster was “General function prediction only”, this followed by “Carbohydrate transport and metabolism”, “Transcription”, and “Amino acid transport and metabolism”.

**Figure 3:**
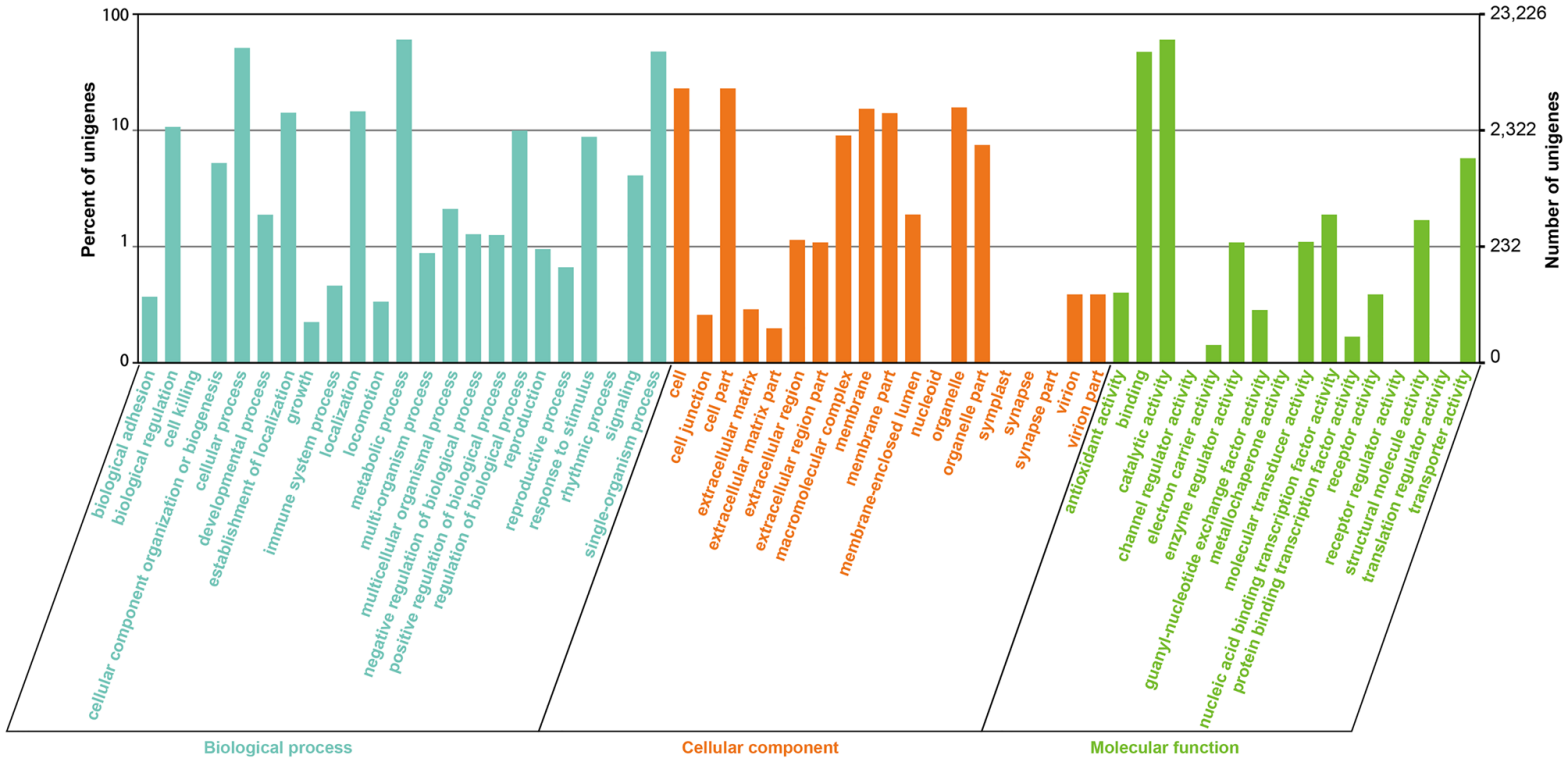
Gene Ontology (GO) classification of *S. latifolia*. The histogram of GO annotations was generated by in-house Perl and R scripts. The unigenes were classified into three main GO categories: ‘Cellular component’, ‘Molecular function’, and ‘Biological process’. On the right Y-axis is the number of unigenes in each category, while on the left Y-axis is the percentage of a specific category. Each unigene could be assigned with more than one GO term.

Overall, 23,226 protein coding unigenes could be classified into 58 GO groups (Fig. 3 and Dataset 3). Among them, there were 23, 19, and 16 groups involved in the biological process, cellular component, and molecular function categories, respectively. The GO terms “cellular process”, “metabolic process”, and “signal-organism process” accounted for the largest proportion subsumed under biological process. Gene numbers involved in “cell”, “cell part”, “organelle”, and “membrane”, were the most abundant under cellular component. The GO terms “binding”, “catalytic activity”, and “transporter activity” encompassed most of the genes under molecular function. In addition, there were 32,765 unigenes that could be mapped to 297 reference canonical KEGG pathways (Dataset 2).

### Analysis of DEGs in the three developmental stages

**Figure 4:**
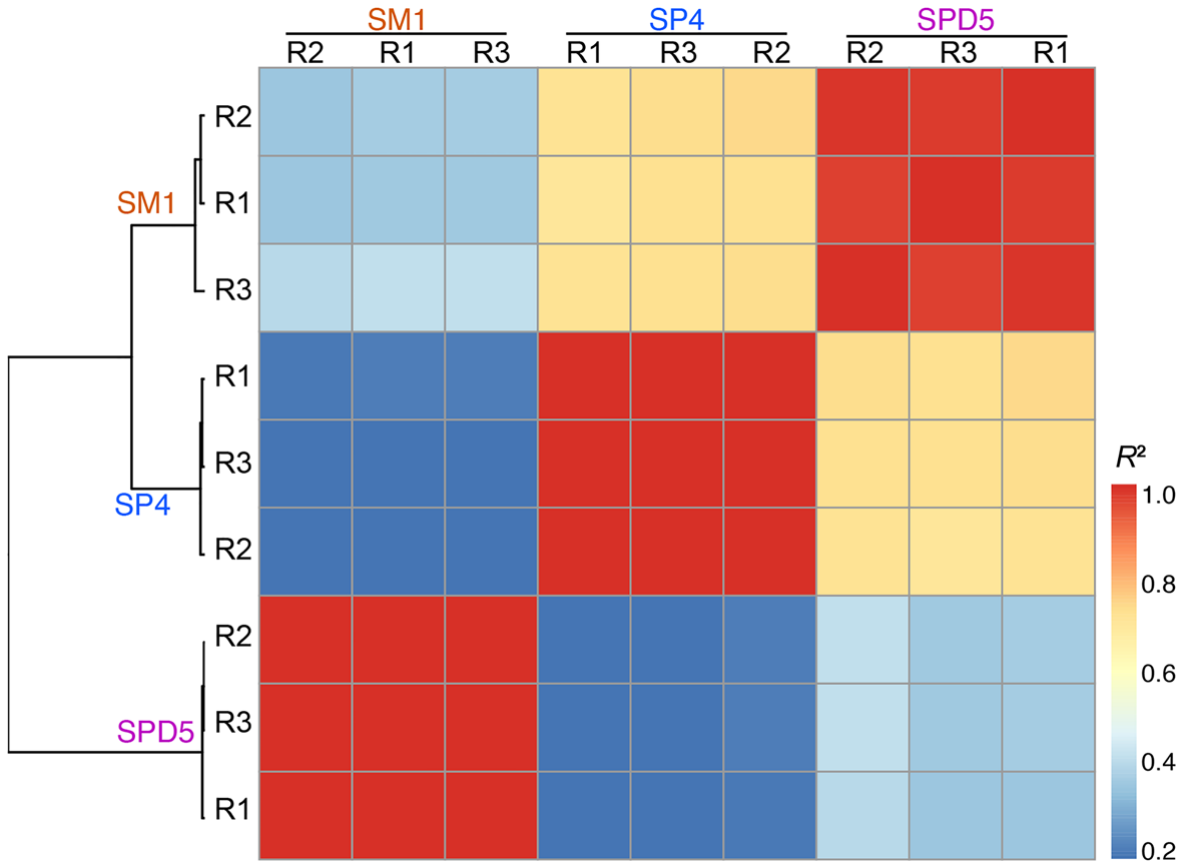
Clustering of samples using FPKM values of all unigenes. Hierarchical clustering of samples was performed using FPKM values of sense transcripts from all derived gene models. The trees were depicted with the ‘cor’ package for R, by using the average linkage distance measurement and Pearson’s correlation.

To determine the candidate genes associated with fruiting body formation, the transcriptome of the SM, SP, and SPD stages were compared for pairs of adjacent developmental stages. Correlation analysis revealed that all sample replicates for each stage clustered together into a single clade (Fig. 4). A total of 8,822 DEGs between SM and SP were identified, these consisting of 5,195 up-regulated and 3,627 down-regulated genes (Fig. 4 and Dataset 4). There were 568, 134, and 21 unigenes respectively assigned to the “Oxidation–reduction process”, “Chromatin binding”, and “MAP kinase tyrosine phosphatase activity”; the expression of these DEGs were all up-regulated during the transition from SM to SP (Fig. S4). The main GO terms of the DEG sets were enriched in “poly(A) RNA binding”, viral transcription”, “nuclear-transcribed mRNA catabolic process”, and “SRP-dependent co-translational protein targeting to membrane” (Table S3; Dataset 5). KEGG enrichment analysis indicated these DEGs were markedly enriched in 40 pathways (FDR( false discovery rate) < 0.01), including “ribosome”, “systemic lupus erythematosus”, “metabolism of xenobiotics by cytochrome P450″, and “ubiquitin mediated proteolysis”*,* among others (Table S4, Dataset 6).

From the SP to SPD phase, a total of 1,347 DEGs were identified, these comprising 690 up-regulated as well as 657 down-regulated genes (Dataset 7). These DEGs were considerably enriched in 251 GO terms, including “protein binding”, “extracellular region”, “extracellular space”, “transport”, and “cell surface” (Dataset 8), and also enriched in 44 KEGG pathways (*FDR* < 0.01), such as “Complement and coagulation cascades”, “Glutathione metabolism”, “Tyrosine metabolism” (Dataset 9).

### Expression changes of genes and pathways involved in fruiting body formation

**Figure 5:**
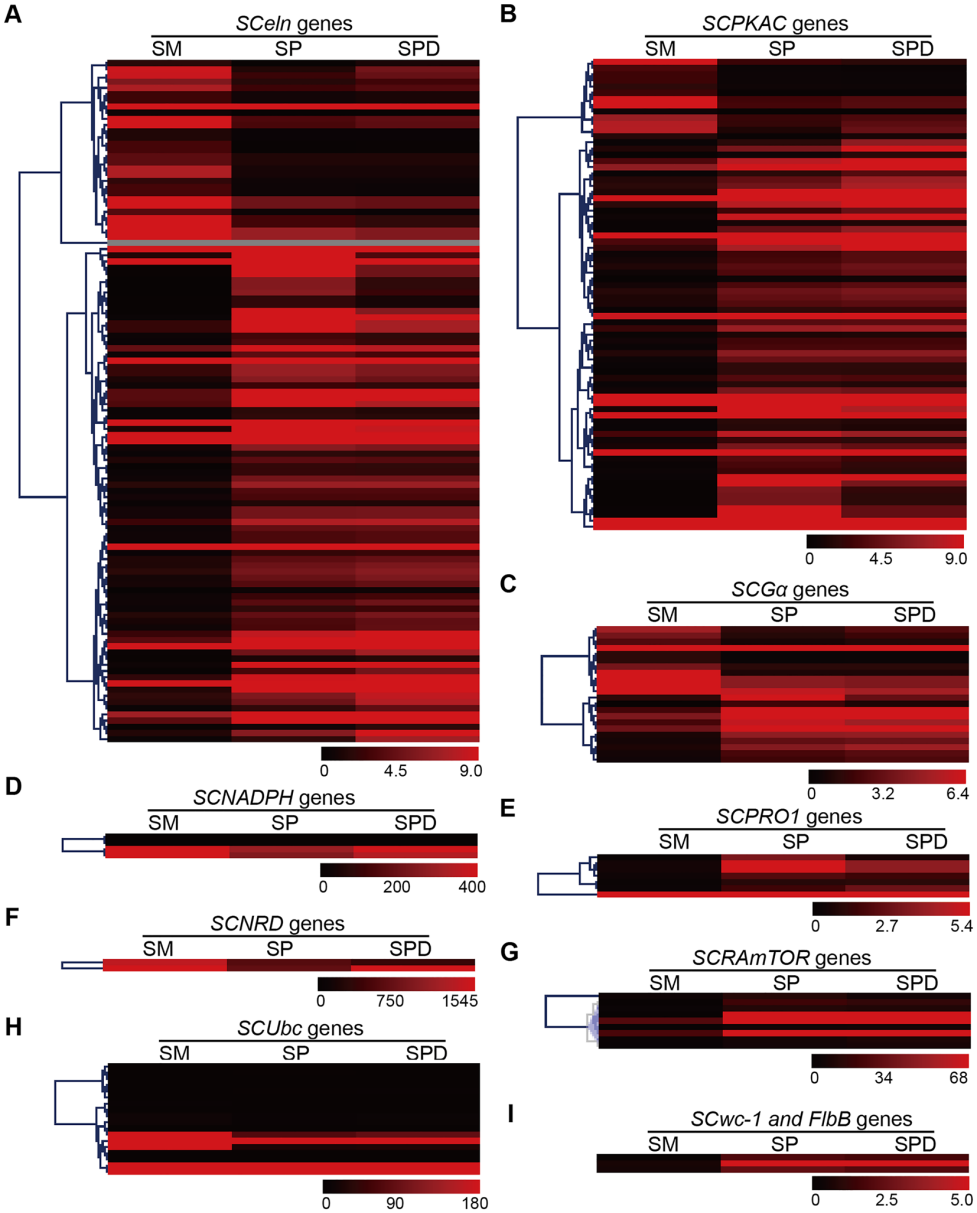
Heat map of important DEGs (differentially expressed genes) likely involved in fruiting formation. (**A**) *SCelen* genes; (**B**) *SCPKAC* genes; (**C**) *SCGα* genes; (**D**) *SCNADPH* genes; (**E**) *SCPRO1* genes; (**F**) *SCNRD* genes; (**G**) *SCRAmTOR* genes; (**H**) *SCUba* genes; (**I**) *SCwc-1* and *FlbB* genes.

The RNA-Seq data was further accessed by examining the expression levels of those genes involved in fruiting body formation that had been identified earlier. The *eln2* gene functions as an positive regulator in the elongation of primordium^18^. Here, there were 83 *eln2-like* transcripts we identified in the *S. latifolia* transcriptome by using the blast tools with the E-value < 1e^–5^ and shared sequence identity more than 60%. For most of these genes, their expression was up-regulated in the SP stage (Fig. 5A), and some of them were up-regulated in the SM phase, which suggested the functional diversification of *eln2* genes. Many important fruiting body formation-related genes, such as *NADPH*, *PKAC*, *PRO*, *UBC2*, and *WC-1*, were significantly changed during the transformation of different stages (Fig. 5).

The MAPK pathway was significantly changed in the transition from SM to SP (Fig. 5B, Fig. S8). The Pkc (CL5150_Contig2_All), Mkk1_2 (CL4664_Contig1_All), Pbs2 (CL2325_Contig1_All), and as well as others, were all up-regulated in the transition from SM to SP. Both the PI3K-Akt signaling pathway and mechanistic target of rapamycin (mTOR) signaling pathway were found significantly up-regulated during the development of SP to SPD (Figs. S9 and S10). Further, those genes encoding 3-phosphoinositide-dependent protein kinase 1 (PDKs, CL4393_Contig11_All, Unigene2284_All, CL4393_Contig13_All, CL4393_Contig16_All, CL4393_Contig5_Al), mTOR (Unigene6719_All, CL458_Contig9_All, Unigene6718_All), serum and glucocorticoid-regulated kinase (SGKs, CL1207_Contig2_All, CL1207_Contig1_All), were also up-regulated during the development of the SP and SPD stages. Lighting is perhaps important for inducing primordium initiation and fruiting body maturation^39^, and indeed both photosynthesis and phototransduction pathways were detected in our annotation. Yet there was no significant enrichment for light-related pathways, which suggested that photo stimulation may less important for *S. latifolia*’s fruiting body formation that that of other mushroom fungi.

### Validation of DEGs by qRT-PCR

A total of 27 genes, including those associated with fruiting body formation, the MAPK pathway, the PI3K-Akt and mTOR signaling pathways, were validated by the qRT-PCR analyses and the expression (Figs. S5–S7). Our results from the qRT-PCR and RNA-Seq data matched up well with each other, which indicated the good quality of the RNA-seq results.

## Discussion

*Sparassis latifolia* is a precious highly-prized mushroom type, mostly because of its medicinal properties^40,41^. Previous research on *S. latifolia* has mainly focused on ways to improve its medical applications^42,43^ and mushroom production^44,45^. Surprisingly little is actually understood mechanisms of its fruiting body’s growth process, as well as the production of medically relevant bioactive metabolites, chiefly because genomic information for *S. latifolia* is unavailable. Our study provided comprehensive cDNA sets that will certainly be useful in further investigations of this species, especially for identifying the candidate genes associated with the fruiting body formation in *S. latifolia* or other edible mushroom species. Moreover, this data can benefit the phylogenetic, diversity, and functional research of other species of this genus and their affinity.

Fruiting body development is often induced by accompanying its living condition which is substantially changed^46^. The transfer of environmental stress signals to cells via the MAPK pathways plays an essential roles in diverse intracellular signaling processes in both plants and fungi^47^. There are many orthologs of MAP kinases gene families which are involved in hyphal and sporocarp growth in fungi^48,49^. In the fruiting body formation of *S. latifolia*, this pathway showed significantly up-regulation during both SP and SPD development (Figs. S4 and S8), and this was supported by the GO and KEGG enrichments (Datasets 5, 6 and 8, 9), which agreed with previous findings reported for *C. cinerea* as well as *H. marmoreus*^25,28^*.*

Not only physiological conditions but also nutritional conditions could substantially influence the process of fruiting body formation in fungi^50^. On the hand, a nitrogen-deficient condition could sustain the basic growth and development of fungi; on the other, it could avoid microbial competition^51,52^. The nitrate reductase gene (Unigene6931_All) was up-regulated in SM and SPD by more than tenfold that in SP (Fig. 5), which suggests development of SM and SPD in *S. latifolia* was promoted by the nitrate starvation condition. In *C. cinerea*, the mTOR signaling pathway was considered to function as a necessary regulator throughout primordium formation^25^. Generally, the mTOR pathway and its associated regulated pathway PI3K-Akt were expressed more during SM, SP, and SPD of *S. latifolia*. These results suggested that continual perception of environmental change might be more important for the later stages of fruiting body formation than those that occur in its early developmental stages. Lastly, other pathways, such as those of “Ribosome”, “Systemic lupus erythematosus”, and “Metabolism of xenobiotics by cytochrome P450” (among others), also varied continuously as the above key single pathways underwent changes in their activity.

## Conclusion

Despite *S. latifolia* now being a globally important edible and medical mushroom species, the molecular or genetic basis of its fruiting body formation and medical value remains largely uncharacterized, especially given the enormous difficulties inherent to the cultivation of cauliflower mushroom. The present work can inform future research work seeking to investigate in-depth the initiation of the fruiting body. In the transition from SM to SP, and then to SPD, environmental signals’ sensing might be involved in primordium initiation as well as fruiting body development. Further functional analysis of the important candidate genes may lead to an enhanced understanding of the network regulating how the fruiting body develops. The crucial candidate genes mapped here to the MAPK, PI3K-Akt, and mTOR signaling pathways offers a convenient starting point for elucidating the molecular or genetic mechanism of fruiting body formation in *S. latifolia*.

## Supplementary Information

Supplementary Information 1.

Supplementary Information 2.

Supplementary Information 3.

Supplementary Information 4.

Supplementary Information 5.

Supplementary Information 6.

Supplementary Information 7.

Supplementary Information 8.

Supplementary Information 9.

Supplementary Information 10.

Supplementary Information 11.

Supplementary Information 12.

Supplementary Information 13.

Supplementary Information 14.

Supplementary Information 15.

Supplementary Information 16.

Supplementary Information 17.

Supplementary Information 18.

Supplementary Information 19.

Supplementary Information 20.

## Data Availability

We have deposited the primary data underlying these analyses as follows: SubmissionID: SUB10980832; BioProject ID: PRJNA799466; BioSample accessions: SAMN25174015; https://submit.ncbi.nlm.nih.gov/subs/sra/SUB10980832/overview.
