# Supplementary figures and images for: De novo transcriptome assembly and comprehensive assessment provide insight into fruiting body formation of Sparassis latifolia
Source: Sci Rep. 2022 Jun 30;12:11075. doi: 10.1038/s41598-022-15382-5 (PMC9247108; doi:10.1038/s41598-022-15382-5)

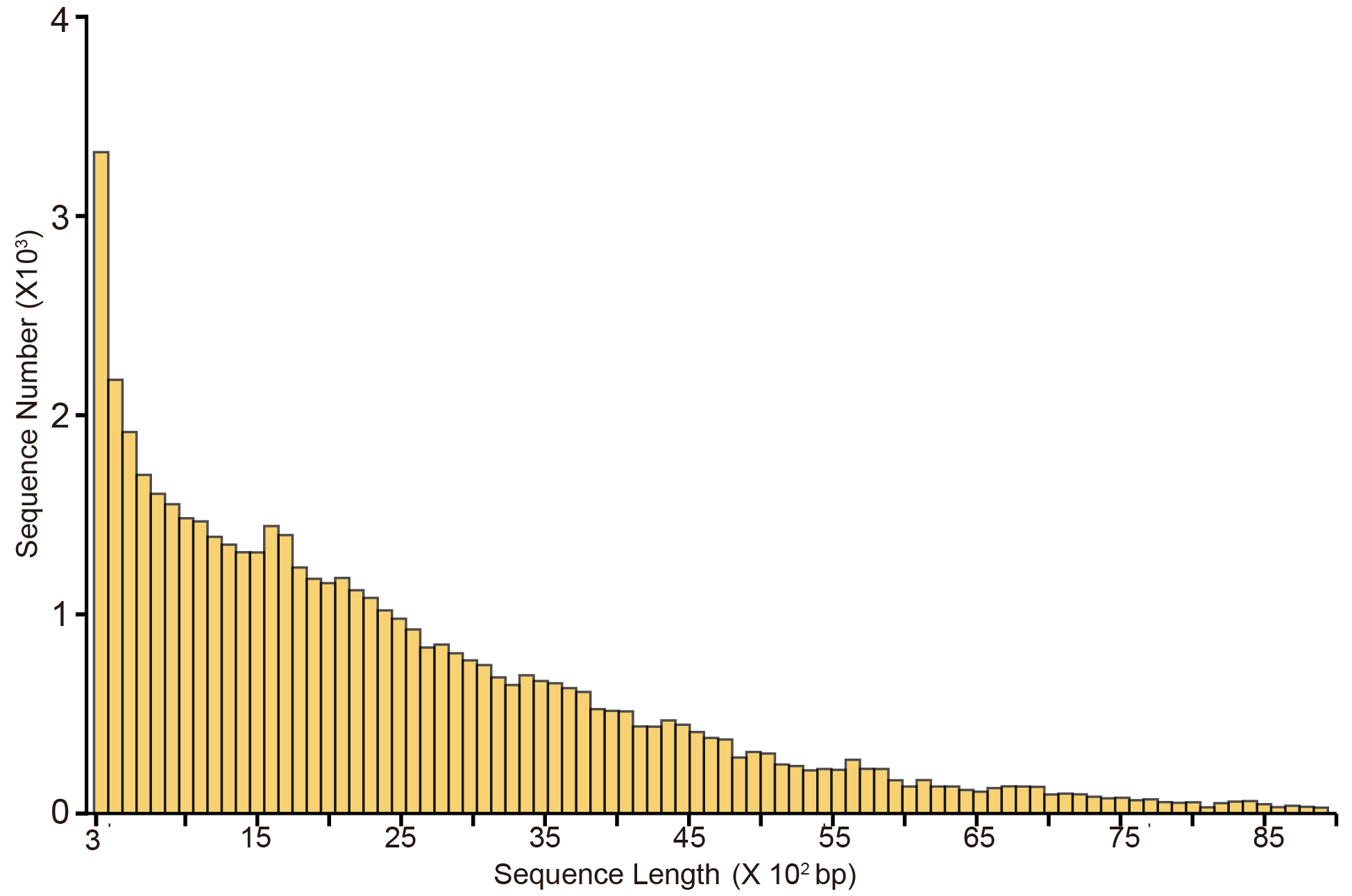

Supplement: Supplementary file 10 — Supplementary Information 10. [file 41598_2022_15382_MOESM10_ESM.png]

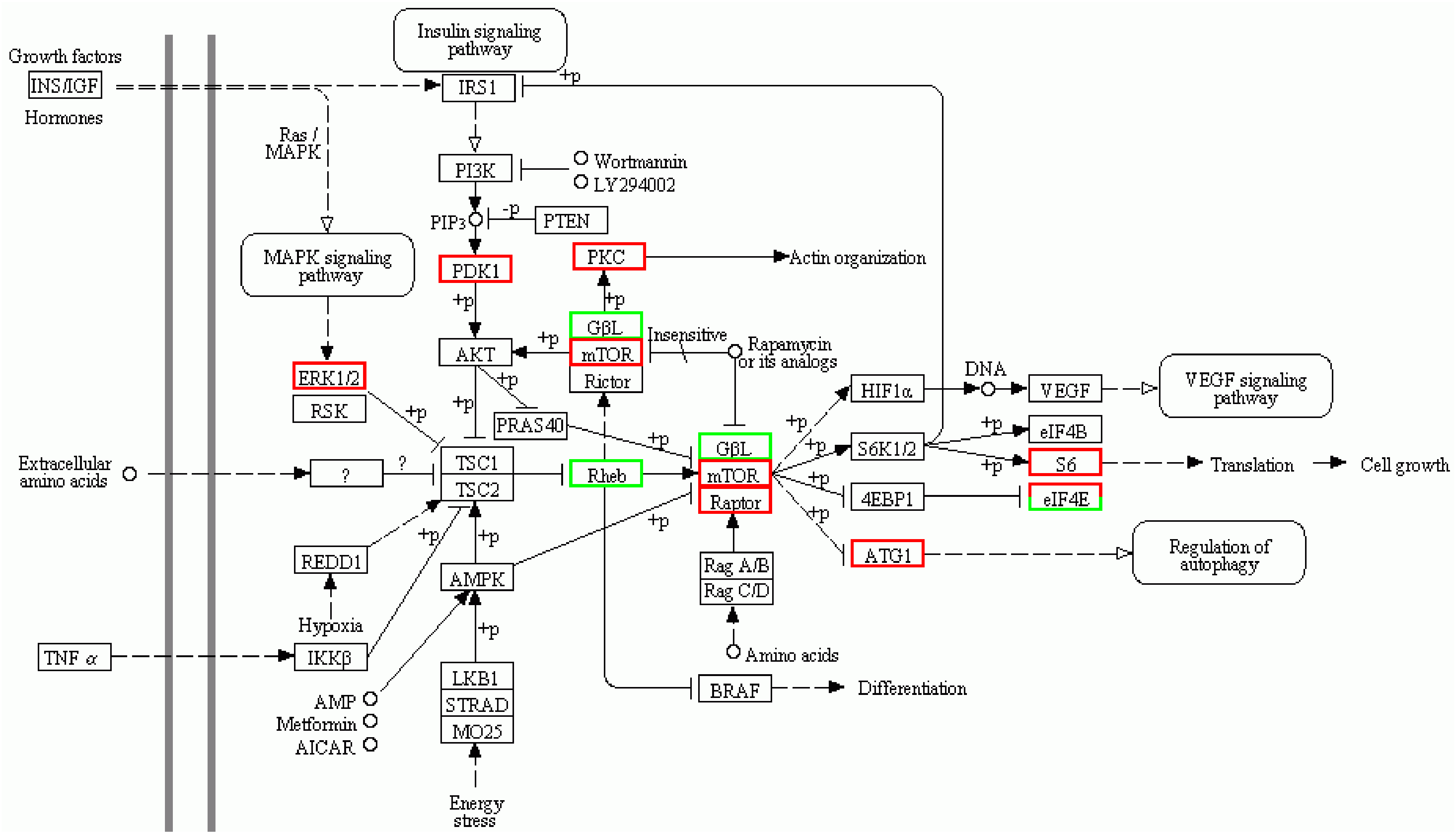

Supplement: Supplementary file 11 — Supplementary Information 11. [file 41598_2022_15382_MOESM11_ESM.png]

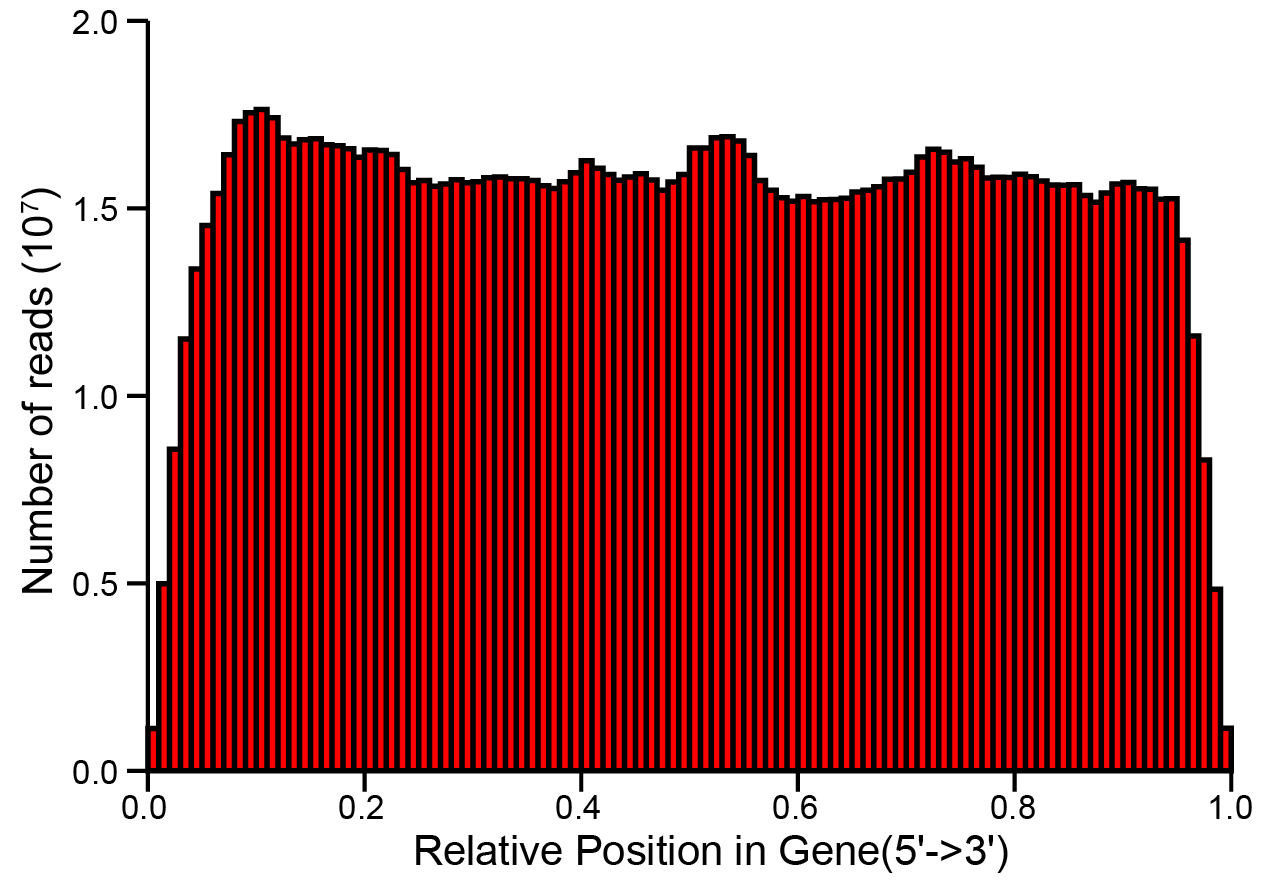

Supplement: Supplementary file 12 — Supplementary Information 12. [file 41598_2022_15382_MOESM12_ESM.png]

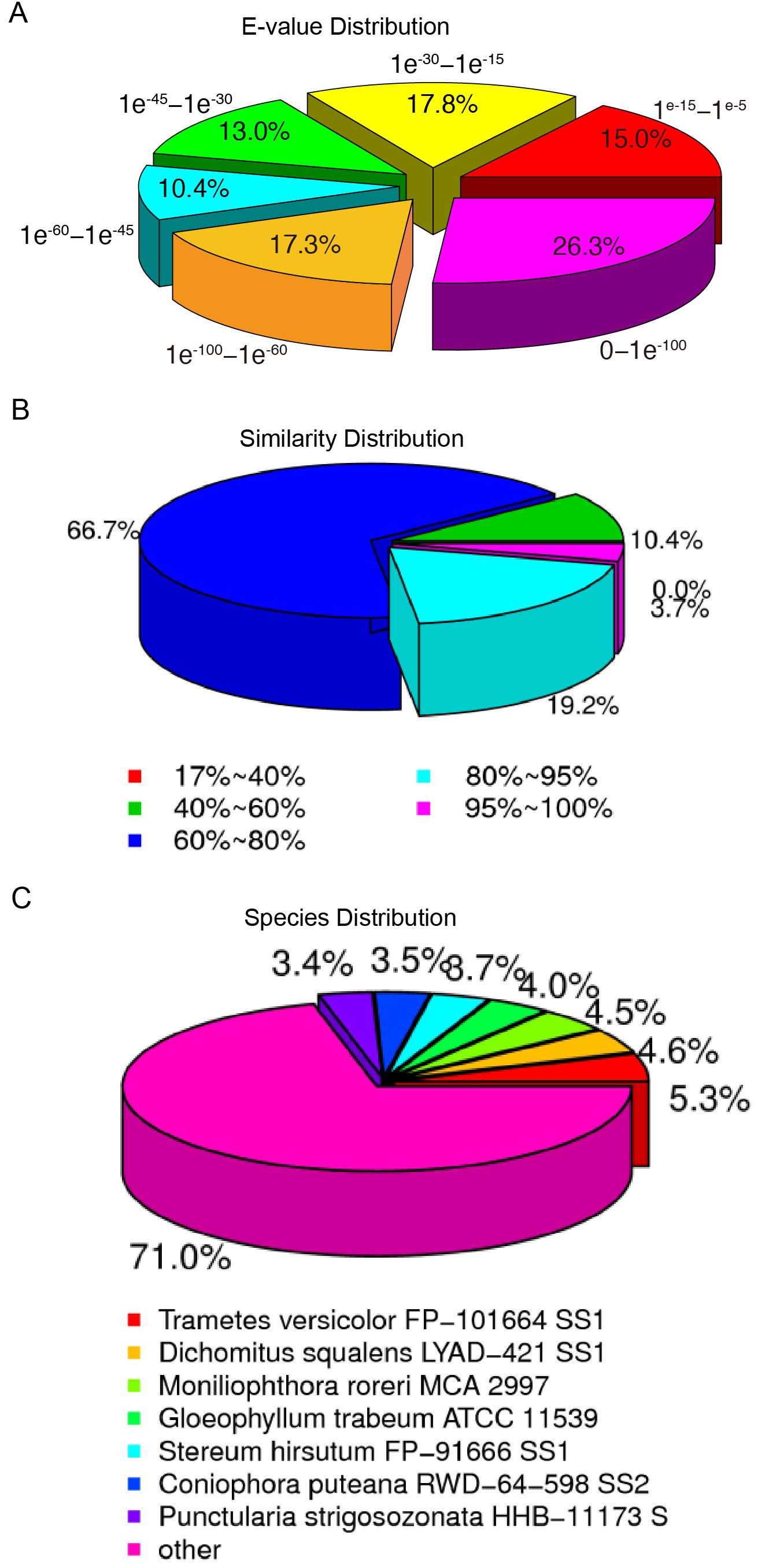

Supplement: Supplementary file 13 — Supplementary Information 13. [file 41598_2022_15382_MOESM13_ESM.png]

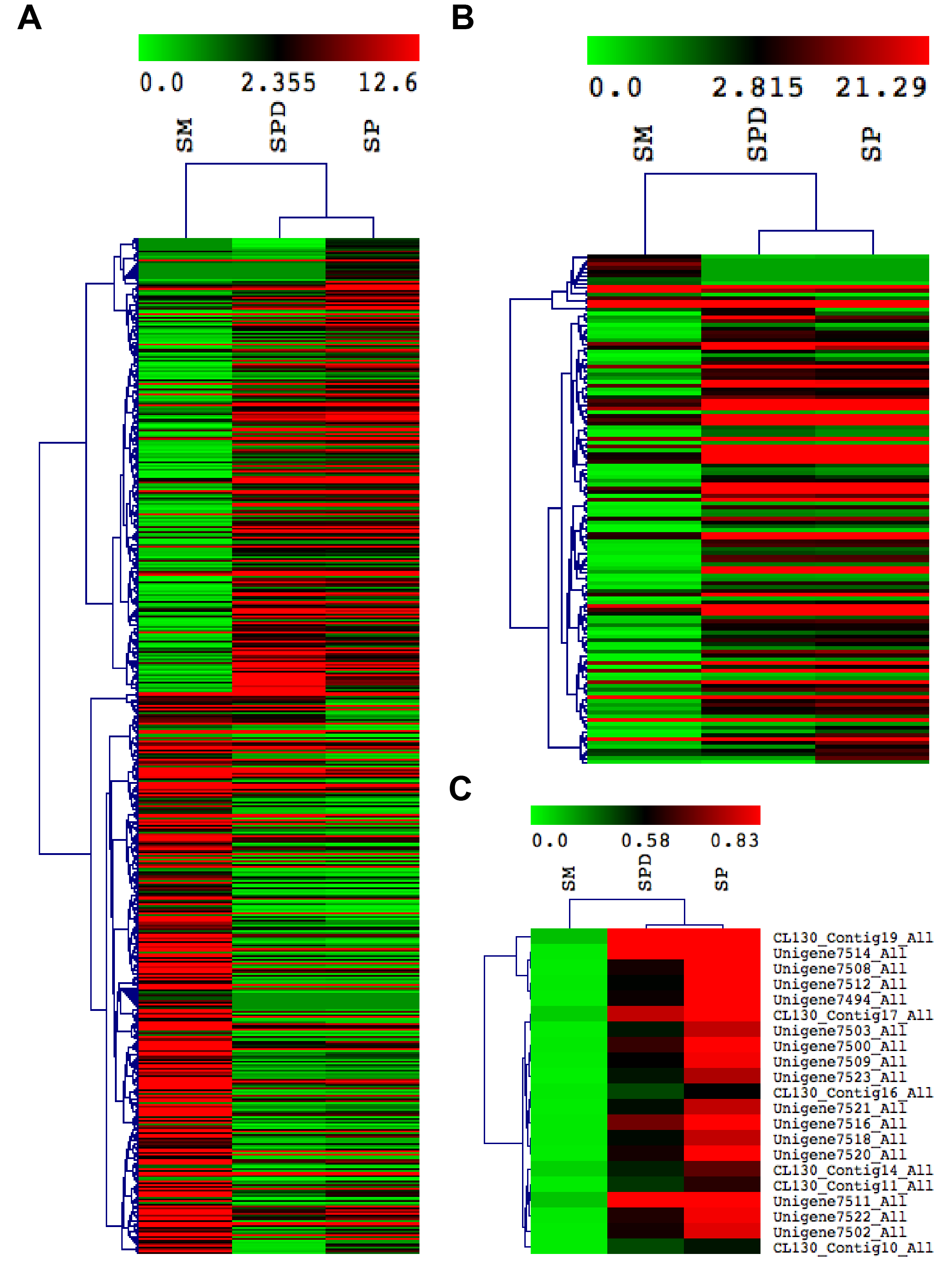

Supplement: Supplementary file 14 — Supplementary Information 14. [file 41598_2022_15382_MOESM14_ESM.png]

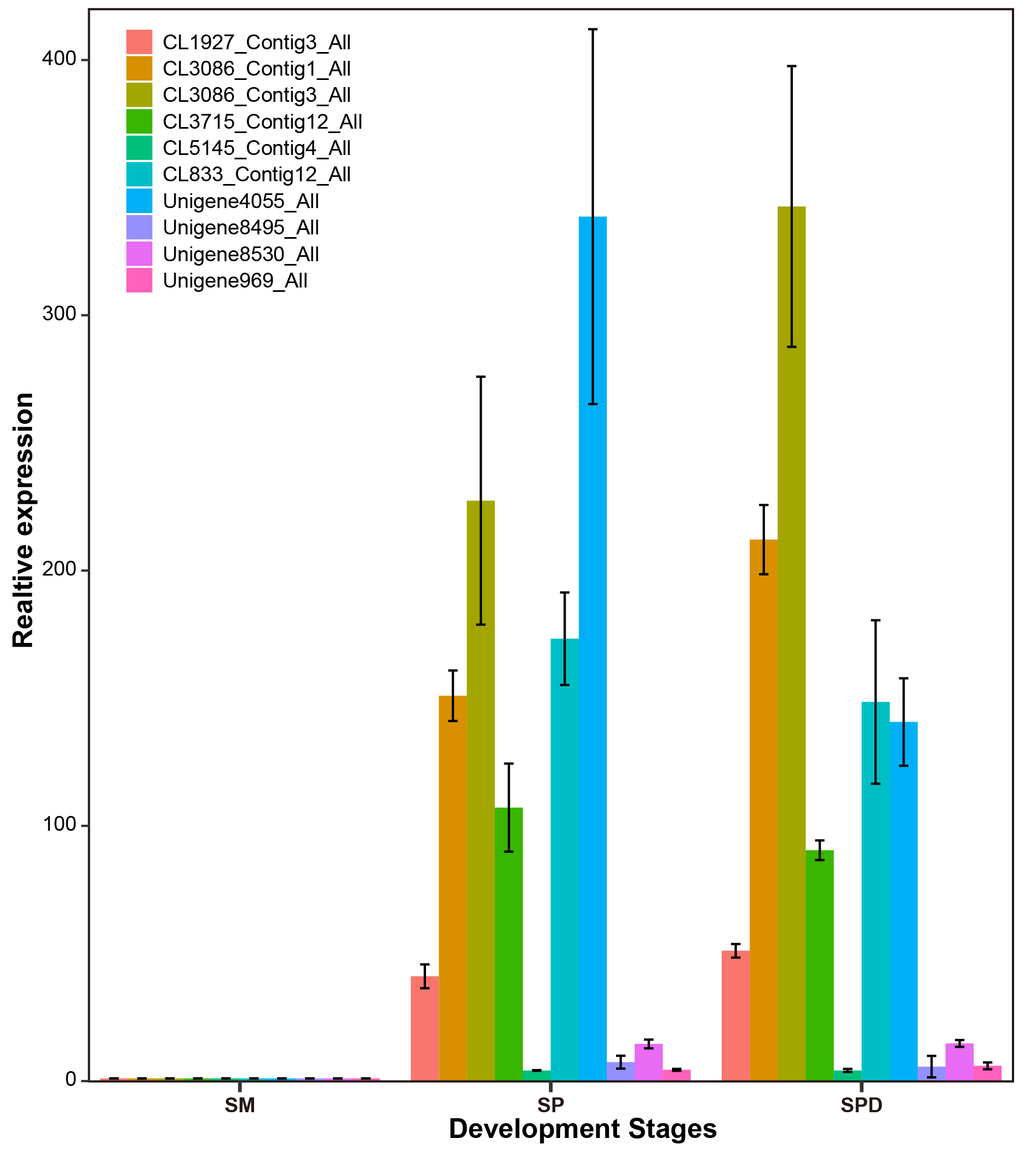

Supplement: Supplementary file 15 — Supplementary Information 15. [file 41598_2022_15382_MOESM15_ESM.png]

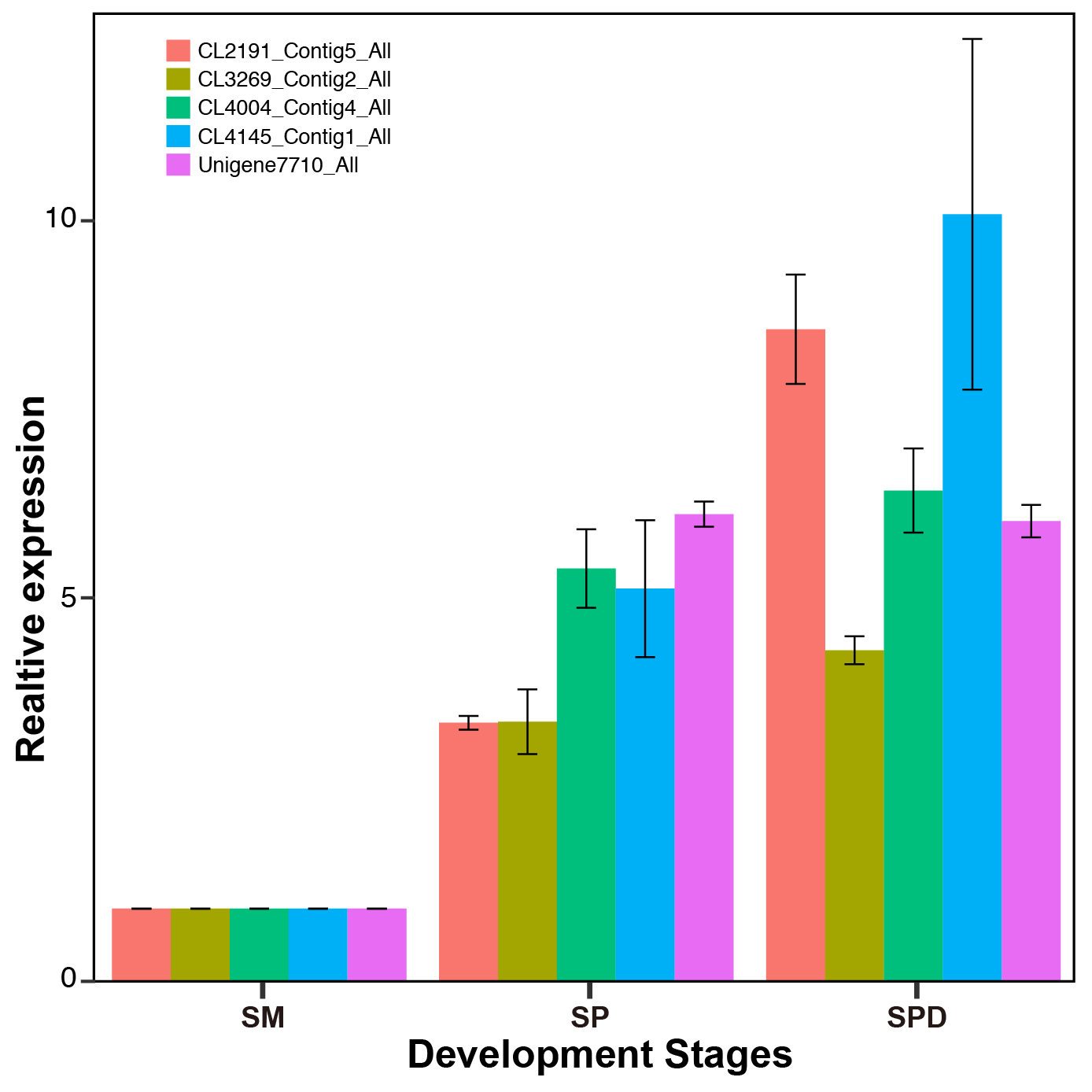

Supplement: Supplementary file 16 — Supplementary Information 16. [file 41598_2022_15382_MOESM16_ESM.png]

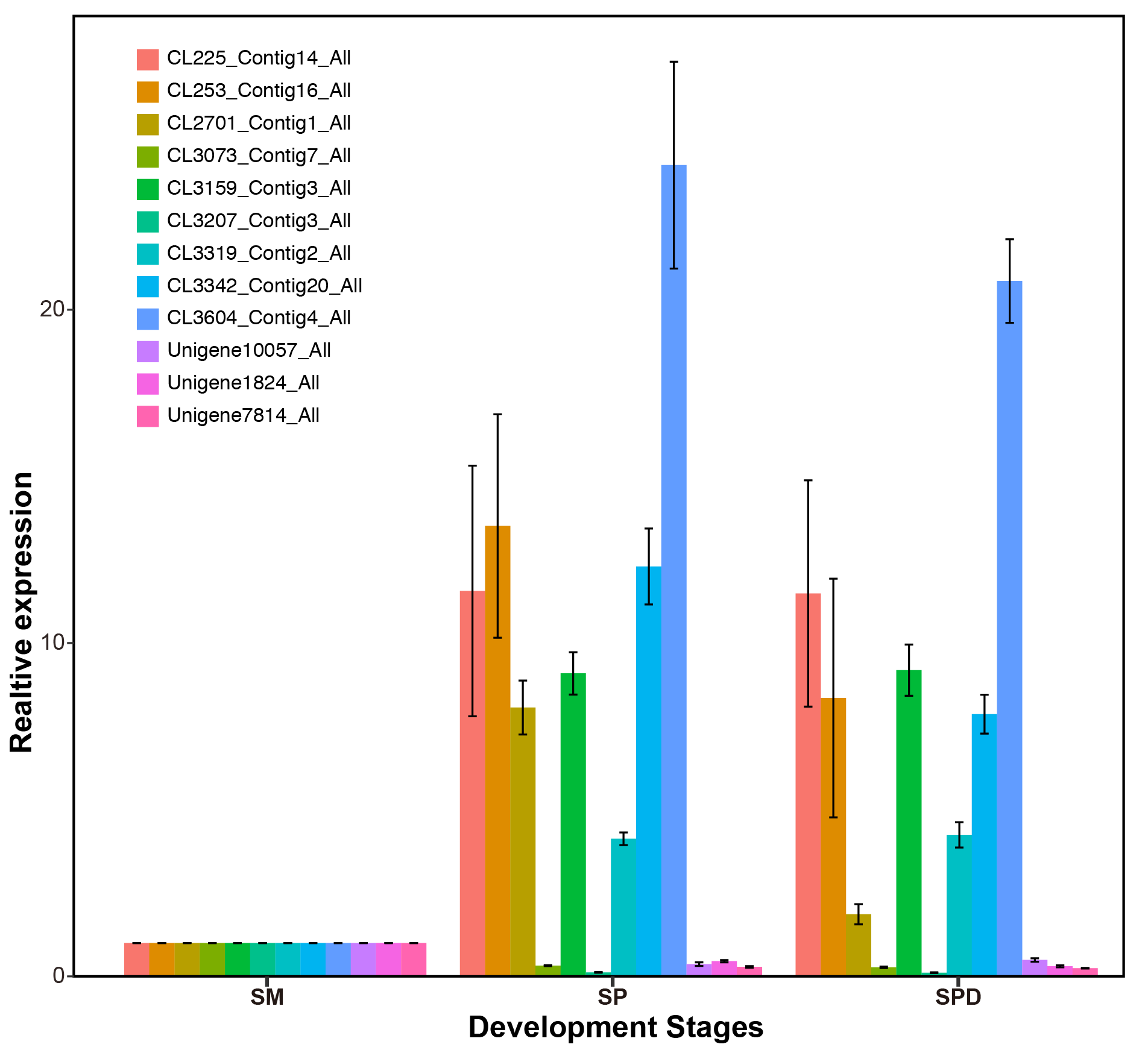

Supplement: Supplementary file 17 — Supplementary Information 17. [file 41598_2022_15382_MOESM17_ESM.png]

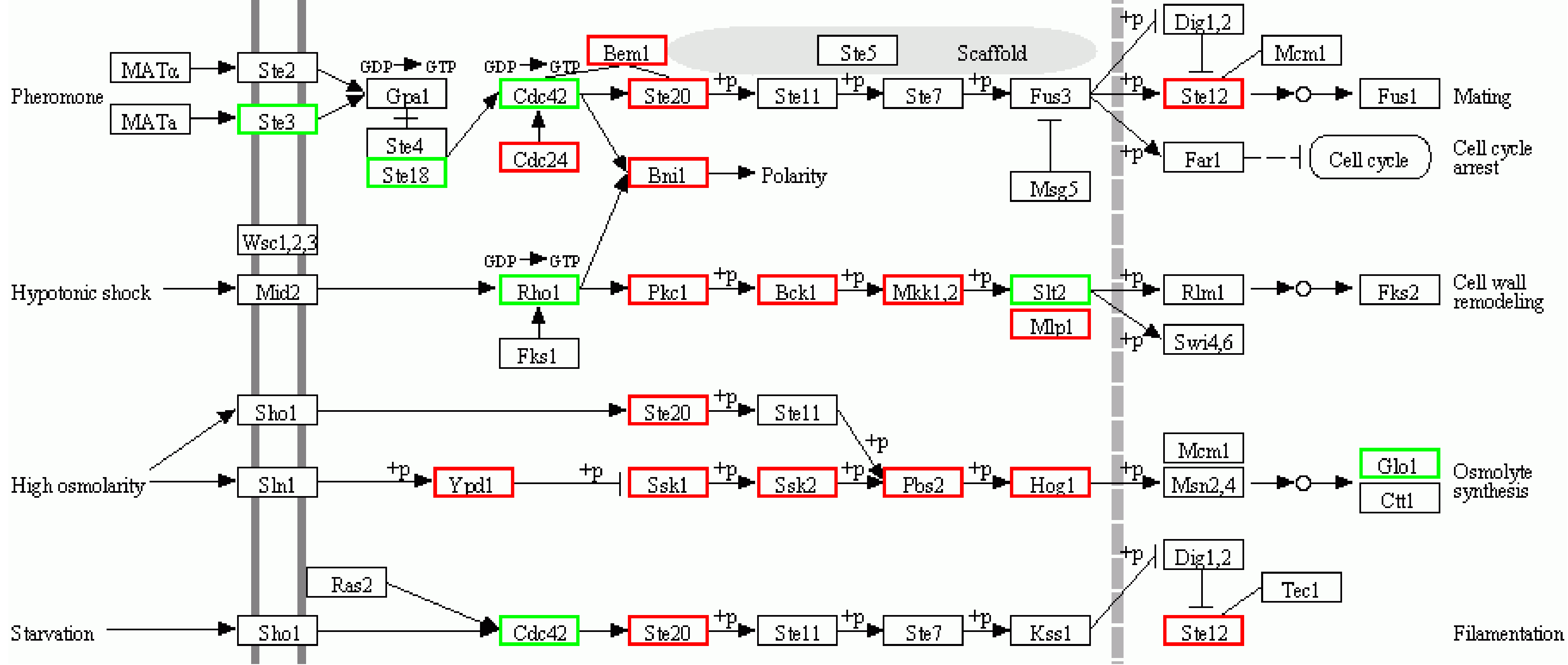

Supplement: Supplementary file 18 — Supplementary Information 18. [file 41598_2022_15382_MOESM18_ESM.png]

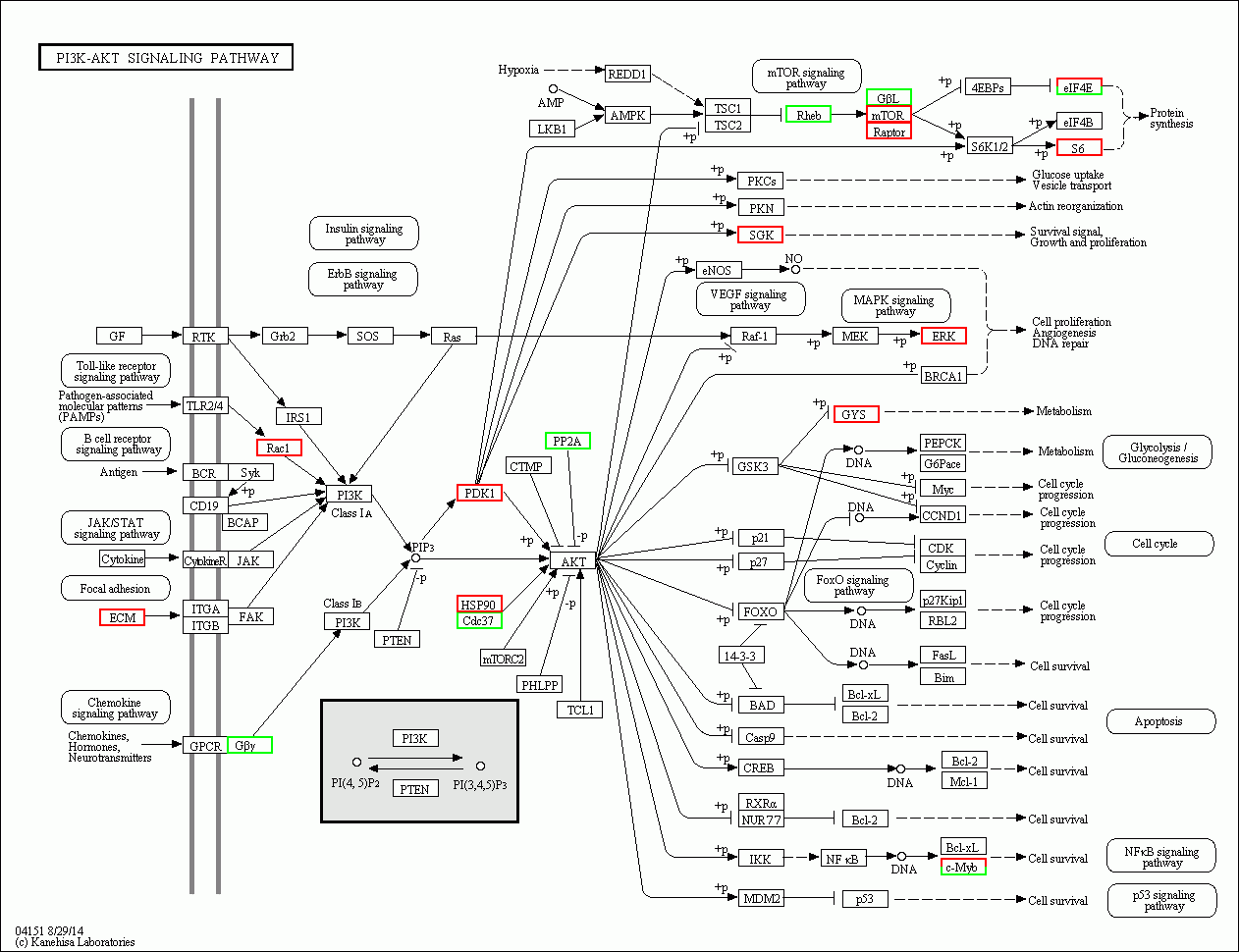

Supplement: Supplementary file 19 — Supplementary Information 19. [file 41598_2022_15382_MOESM19_ESM.png]
