# Supplementary material for: De novo transcriptome assembly and comprehensive assessment provide insight into fruiting body formation of Sparassis latifolia
Source: Sci Rep. 2022 Jun 30;12:11075. doi: 10.1038/s41598-022-15382-5 (PMC9247108; doi:10.1038/s41598-022-15382-5)
Supplement: Supplementary file 20 — Supplementary Information 20. [file 41598_2022_15382_MOESM20_ESM.docx]

***De novo* transcriptome assembly and comprehensive assessment** **provide insight into fruiting body formation of *Sparassis latifolia***

Lili Shu^1^, Miaoyue Wang^1^, Hui Xu^1^, Zhiheng Qiu^1*^ and Tianlai Li^1*^

^1^School of Horticulture, Shenyang Agricultural University, Shenyang,110866, China.

*Corresponding author: Zhiheng Qiu and Tianlai Li

Correspondence: qiuzh@syau.edu.cn & tianlaili@126.com

**Supplementary Information**

**Figure S1.** The length distribution of assembled *S.* *latifolia* unigenes.

**Figure S2.** The reads coverage of all final unigenes of *S.* *latifolia*.

**Figure S3.** Classification of NR annotation results of the *S.* *latifolia* transcripts. (**A**) E-value distribution, (**B**) Similarity distribution, and (**C**) Species distribution.

**Figure S4.** Heat map analysis of three significantly up-regulated GO annotation gene sets. (**A)**, (**B)**, and (**C)** represent the gene sets of “Oxidation-reduction process”, “Chromatin binding”, and “MAP kinase tyrosine phosphatase activity”, respectively.

**Figure S5.** The qRT-PCR validation of the gene set *eln2*s.

**Figure S6.** The qRT-PCR validation of the gene set *PAKC*s.

**Figure S7.** The qRT-PCR validation of *Gα*, *NADPH*, *NDR*, *PRO1*, *mTOR*, *UBC2*, and *WC-1* genes.

**Figure S8.** The MAPK pathway identified by the KEGG annotation analysis. The pathway was produced online (at http://www.kegg.jp/keggbin/show_pathway? ko00980). The up-regulated genes are indicated with red boxes and the down-regulated genes by green boxes.

**Figure S9.** The PI3K-Akt pathway identified by the KEGG annotation analysis. The pathway was produced online (at http://www.kegg.jp/keggbin/show_pathway?ko04151). The up-regulated genes are indicated with red boxes and down-regulated genes by green boxes.

**Figure S10.** The mTOR pathway identified by the KEGG annotation analysis. The pathway was produced online (at http://www.kegg.jp/keggbin/show_pathway?ko04150). The up-regulated genes are indicated with red boxes and down-regulated genes by green boxes.

**Dataset 1.** Nr database annotation results of all unigenes of *S. latifolia*.

**Dataset 2.** The expression and annotation of all unigenes in the three development stages.

**Dataset 3.** The GO annotation of all unigenes.

**Dataset 4.** The sets of DEGs in the transition from SM to SP.

**Dataset 5.** GO enrichment results of the sets of DEGs in the transition from SM to SP.

**Dataset 6.** KEGG enrichment results of the sets of DEGs in the transition from SM to SP.

**Dataset 7.** The sets of DEGs in the transition from SP to SPD.

**Dataset 8.** GO enrichment results of the sets of DEGs in the transition from SP to SPD.

**Dataset 9.** KEGG enrichment results of the sets of DEGs in the transition from SP to SPD.

| **Gene name** | **Primer F (5’-3’)** | **Primer R (5’-3’)** | **Unigene ID** |
| --- | --- | --- | --- |
| *18sRNA* | GCGCAAGTACTCTGTGTGGA | GAGGGGCCTGACTCATCATA | CL4182_Contig1_All |
| *Sceln2-1* | ATTGTTATTTGCGGCCAAAG | GGCGTAGCTTCTTCAACCAG | CL1927_Contig3_All |
| *Sceln2-2* | CACCACTCGGAGGACAGAAT | AGTGAGCCACGCCTACAGTT | CL3086_Contig1_All |
| *Sceln2-3* | CACCACTCGGAGGACAGAAT | AGTGAGCCACGCCTACAGTT | CL3086_Contig3_All |
| *Sceln2-4* | TATTCGCTCCCGTACTCGTT | GCGGATTTCTGCTTCAACTC | CL5145_Contig4_All |
| *Sceln2-5* | AGTCGGGACGAGAAGGTGTA | TCGCCTTGAAGTATGGCTCT | CL3715_Contig12_All |
| *Sceln2-6* | ATCTTCCACATCTGGGCATC | GCCATGACATTGGGGATTAC | CL833_Contig12_All |
| *Sceln2-7* | CATGATGGTGTGCTGGATTC | GTGGGTGCACAGGAGGTTAT | Unigene8530_All |
| *Sceln2-8* | CCCATCCTCAGGAATCTTCA | AGTTGAGCTCGTGGTGGAGT | Unigene969_All |
| *Sceln2-9* | CGTTTGATCCCAGCTACACA | CATCTTCTGCCGCTACAACA | Unigene8495_All |
| *Sceln2-10* | GTGCTTCGCCTGAACTTTTC | CTTGTGCGAATTGAGGATGA | Unigene4055_All |
| *ScGα-1* | GTCCGAAAACATCGGAAAGA | GGCTGAAGTTGGAGGAGACA | CL2701_Contig1_All |
| *ScGα-1* | CTGTAAATCGCGAACGTCCT | GTCGTTGCCGGTAGTTCATT | CL3073_Contig7_All |
| *ScGα-3* | GGGATCAAAGTCCGCAAATA | TGTGCACCTGGATGAAGTGT | CL3207_Contig3_All |
| *ScPKAc-1* | TCAGCAGAGTAGAGCGACGA | GCAGGAAGGTCACGTTTTGT | CL2191_Contig5_All |
| *ScPKAc-2* | ACACCGATAGATCGGACCAG | CGAGGAAATGAGTGTGAGCA | CL3269_Contig2_All |
| *ScPKAc-3* | CCGTTCTCCTTTCCTCTGTG | GAGGGCATGCTACCGAATAA | CL4004_Contig4_All |
| *ScPKAc-4* | TCACAAGTTCAGGTCGCTTG | CCGCTTATCACCTTCACGTT | CL4145_Contig1_All |
| *ScPKAc-5* | GACTGGTTCTCCTGCTGGTC | TTCCCCACCCAGAACAATTA | Unigene7710_All |
| *ScRAmTOR-1* | CCGGATAGTTCGTGAGGTGT | GATGCTCCACTGCTCGTACA | CL3319_Contig2_All |
| *ScRAmTOR-2* | ACAACAGGAGGGTGATCTGC | ATCACCTAAAACGGGGAACC | CL3604_Contig4_All |
| *ScUBC2-1* | CGATCTCGAATCCTTCCGTA | GGAGGGCCGTAGATTTTCTC | CL225_Contig14_All |
| *ScUBC2-2* | CTGCAGAATATCGAGGCACA | ACGAGTCGTACCCGAACAAG | Unigene10057_All |
| *ScUBC2-3* | ACAAGAATGGCACCTTCCAC | TCGTTAATTCCGGGGTGATA | Unigene7814_All |
| *Scwc-1* | GACCGGTCAGACTCGTTGTT | GGCTTGCTCTTGAAGGTGAC | CL3159_Contig3_All |
| *ScNADPH* | TATGGGATTGCGACAACAGA | CCAGTCTCTTCAACGCATCA | CL253_Contig16_All |
| *ScNRD* | ACAAGAGTGGGATGGTGGTC | CGTTGGTCCAATTCACACTG | Unigene1824_All |
| *ScPRO1* | CGTTCGCATGCTACGTTTTA | CGTAGACGGGGTGGTGTAGT | CL3342_Contig20_All |

**Table S1.** Primers used for RT-PCR in this study.

| **Samples** | | **Raw reads (×10^6^)** | **Clean reads (×10^6^)** | **Clean nucleotides (Gb)** | **≥ Q30 (%)** | **GC (%)** |
| --- | --- | --- | --- | --- | --- | --- |
| **Stage** | **Replicate** |  |  |  |  |  |
| SM | 1 | 46.39 | 44.97 | 6.30 | 94.49 | 55.39 |
|  | 2 | 55.07 | 53.39 | 7.47 | 94.27 | 55.50 |
|  | 3 | 56.66 | 54.89 | 7.68 | 94.13 | 55.35 |
| SP | 1 | 57.73 | 55.87 | 7.82 | 93.28 | 55.51 |
|  | 2 | 56.74 | 54.89 | 7.68 | 94.53 | 55.91 |
|  | 3 | 58.96 | 57.00 | 7.98 | 94.12 | 55.48 |
| SPD | 1 | 53.83 | 52.11 | 7.30 | 94.27 | 55.66 |
|  | 2 | 56.11 | 54.18 | 7.58 | 94.17 | 56.19 |
|  | 3 | 56.87 | 54.89 | 7.68 | 93.89 | 55.30 |
| Total | | 498.36 | 482.19 | 67.49 |  |  |

**Table S2.** Summary of raw reads and clean reads of the RNA-Seq.

| **GO ID** | **GO Term** | **Category** | **Gene Number** | **–Log(FDR)** |
| --- | --- | --- | --- | --- |
| 0044822 | Poly(A) RNA binding | MF | 287 | 12.51 |
| 0019083 | Viral transcription | BP | 63 | 12.37 |
| 0000184 | Nuclear-transcribed mRNA catabolic process | BP | 73 | 10.59 |
| 0006614 | SRP-dependent cotranslational protein | BP | 66 | 10.15 |
| 0006412 | Translation | BP | 169 | 9.00 |
| 0005840 | Ribosome | CC | 108 | 8.72 |
| 0006364 | rRNA processing | BP | 164 | 8.63 |
| 0006413 | Translational initiation | BP | 88 | 8.48 |
| 0030529 | Intracellular ribonucleoprotein complex | CC | 105 | 8.02 |
| 0070062 | Extracellular exosome | CC | 390 | 7.95 |
| 0043937 | Regulation of sporulation | BP | 21 | 3.45 |
| 0033550 | MAP kinase tyrosine phosphatase activity | MF | 21 | 3.45 |
| 0005576 | Extracellular region | CC | 303 | 3.41 |
| 0016791 | Phosphatase activity | MF | 48 | 3.32 |
| 0032880 | Regulation of protein localization | BP | 36 | 3.25 |
| 1903385 | Regulation of homophilic cell adhesion | BP | 20 | 3.24 |
| 0008092 | Cytoskeletal protein binding | MF | 30 | 3.24 |
| 0034599 | Cellular response to oxidative stress | BP | 104 | 3.18 |
| 0007165 | Signal transduction | BP | 175 | 3.16 |
| 0005351 | Sugar:proton symporter activity | MF | 14 | 3.10 |
| 0098869 | Cellular oxidant detoxification | BP | 25 | 2.73 |
| 0032040 | Small-subunit processome | CC | 46 | 2.68 |
| 0046323 | Glucose import | BP | 13 | 2.66 |
| 0003682 | Chromatin binding | MF | 134 | 2.65 |
| 0055114 | Oxidation-reduction process | BP | 568 | 2.54 |
| 0005355 | Glucose transmembrane transporter activity | MF | 13 | 2.34 |

**Table S3.** GO enrichment results of DEGs sets of the transition from SM to SP. MF is “Molecular Function”; BP is “Biological Process”; CC is “Cellular Component”.

| **Pathway ID** | **Pathway** | **Gene number** | **–Log(FDR)** |
| --- | --- | --- | --- |
| ko03010 | Ribosome | 123 | 15.33 |
| ko05322 | Systemic lupus erythematosus | 51 | 12.73 |
| ko00480 | Glutathione metabolism | 71 | 3.66 |
| ko03015 | mRNA surveillance pathway | 67 | 3.07 |
| ko05204 | Chemical carcinogenesis | 56 | 2.67 |
| ko05134 | Legionellosis | 27 | 2.35 |
| ko04745 | Phototransduction - fly | 9 | 2.13 |
| ko05133 | Pertussis | 17 | 2.13 |
| ko04612 | Antigen processing and presentation | 15 | 2.09 |
| ko05164 | Influenza A | 31 | 2.08 |
| ko04120 | Ubiquitin mediated proteolysis | 69 | 1.87 |
| ko04610 | Complement and coagulation cascades | 14 | 1.82 |
| ko00190 | Oxidative phosphorylation | 69 | 1.63 |
| ko00040 | Pentose and glucuronate interconversions | 41 | 1.56 |
| ko04011 | MAPK signaling pathway - yeast | 33 | 1.55 |
| ko00982 | Drug metabolism - cytochrome P450 | 63 | 1.36 |
| ko04113 | Meiosis - yeast | 64 | 1.14 |
| ko00531 | Glycosaminoglycan degradation | 7 | 1.14 |
| ko00980 | Metabolism of xenobiotics by cytochrome P450 | 65 | 1.14 |
| ko05162 | Measles | 16 | 1.14 |
| ko04141 | Protein processing in endoplasmic reticulum | 70 | 0.92 |
| ko05012 | Parkinson's disease | 57 | 0.92 |
| ko04510 | Focal adhesion | 36 | 0.88 |
| ko04151 | PI3K-Akt signaling pathway | 51 | 2.62 |
| ko04150 | mTOR signaling pathway | 23 | 2.50 |

**Table S4.** KEGG enrichment results of the sets of DEGs (differentially expressed genes) involved in the transition from SM to SP.
